# Surgical Management of Perianal Crohn’s Disease with the Turnbull–Cutait Procedure: A Case Report

**DOI:** 10.3390/life15030460

**Published:** 2025-03-14

**Authors:** Arda Ulaş Mutlu, Hakan Ümit Ünal, Mehmet Gülmez, Can Saraçoğlu, Erman Aytaç, Murat Saruç

**Affiliations:** 1 Department of General Surgery, Acıbadem University, Istanbul 34638, Türkiye; 2 Department of Gastroenterology, Acıbadem University, Istanbul 34638, Türkiye; 3 Colorectal Surgery Northshore University Hospital Northwell Health, New York, NY 11030, USA; 4 Department of General Surgery, Atakent Acıbadem Hospital, Istanbul 34303, Türkiye

**Keywords:** Crohn’s disease, Turnbull–Cutait, complex fistula

## Abstract

Perianal complications of Crohn’s disease are one of the significant reasons for abdominoperineal resection in patients with medically refractory perianal Crohn’s disease. A 35-year-old man with Crohn’s disease with colostomy presented to our clinic due to perianal fistulas. Complete stricture of the anus was observed, and the patient was found unsuitable for the stoma closure. The Turnbull–Cutait procedure was performed and he was discharged on the fifth postoperative day. In the second month after surgery, rectoscopy revealed a 2 cm long pouch on the neorectum. Then, the patient was treated with antibiotics and infliximab. Following the treatment, the symptoms of the patient were regressed. In the 10th postoperative month, a perianal abscess was seen, and the patient was treated with antibiotics. His ileostomy was reversed in the 18th postoperative month without any complications. In the 42nd postoperative month, no complications were reported. This case report presents the index perianal Crohn’s disease case successfully treated with the Turnbull–Cutait procedure.

## 1. Introduction

Perianal abscesses and fistulation are common complications of CD with perianal involvement, affecting 13–38% of patients with CD [1,2]. More than one-fifth of these patients suffer from perianal fistula at least once in their lifetime, and different modalities can be applied to treat patients according to their clinical presentation [3]. Different medical and surgical approaches can be applied in selective patient groups in cases of anal fistulas. Still, more than 20% of these patients undergo total proctocolectomy and end stoma in refractory cases [4,5].

Anal strictures are another common complication of CD due to transmural inflammation. The diagnosis of anal stricture can be easily established via digital examination. Whilst anal stricture can be asymptomatic, patients may be suffering from pain and incontinence in severe strictures. Severe perianal involvement and rectal stenosis are stronger predictors of permanent diversion and total proctocolectomy, which half of the patients with rectal stricture experience [6,7].

The Turnbull–Cutait procedure is defined by Turnbull Jr. [8] for children and Cutait [9] for adults in the early 1960s. In the first step of this two-step procedure, the affected segment of the colon is resected, and the conduit maintained from the proximal colon is exteriorized through the anal canal. In the second step, the exteriorized segment of the colon is amputated, and coloanal anastomosis is matured [10]. Even though this was a historical procedure, it is being performed again in low rectal tumor surgery to prevent the patient from living with a permanent stoma, a major negative factor affecting patients’ quality of life [10,11]. This procedure is usually used in rectum cancer, re-operative pelvic surgery, or recto-urethral fistulas secondary to radiotherapy.

In our knowledge, this is the index case of a patient with isolated perianal CD and complex anal fistulas treated with TC procedure with midterm follow-up.

## 2. Case

A 35-year-old man with fistulizing CD presented to our clinic due to fistulizing CD with perianal involvement. In his medical history, he was diagnosed in 2008 in his home country, Iran. He was treated with mesalazine for 5 years until he had a perianal fistula in 2013. His fistula was treated surgically, and the patient started to have azathioprine and adalimumab combination therapy. One and a half months after the initiation of the therapy, adalimumab was stopped since the disease was refractory. In the following 7 years, he was operated on multiple times due to recurring complex perianal fistulas and abscesses. Abscesses and fistulas were managed with non-cutting seton placement and abscess drainage. These recurrent interventions resulted in anal stricture and the patient continued to have recurrent fistulas. In August 2020, diverting sigmoid colostomy was performed due to anal stricture and multiple non-healing complex anal fistulas. He continued to have azathioprine for one year. In this period, he did not have any de novo anal fistula or abscess. In May 2021, he was referred to our clinic for the colostomy reversal evaluation. In his examination under anesthesia, complete stricture of the rectum and anal was seen and found not suitable for the stoma closure. The TC procedure was suggested to the patient as an option to the proctectomy with permanent end colostomy.

**Figure 1:**
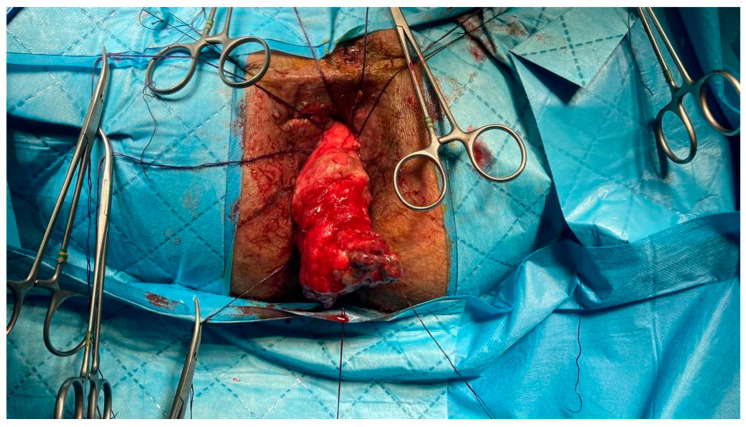
Pulled through conduit maintained from the proximal colon.

The TC procedure was decided, and the patient was operated in July 2021. In the first step, an open approach was preferred. The patient was positioned in the lithotomy position, and transabdominal low anterior resection with total mesorectal excision was completed. Splenic flexure was mobilized, and after guaranteeing the successful pulling through, we got to the perianal phase. In the perianal phase, setons were removed. After completing the mucosectomy, eight 3/0 polyglycolic acid sutures with their needles were placed, enclosing the anal canal to complete the anastomosis in the second step (Figure 1). Back to the abdominal phase, one suction drain was placed in the pelvis. A diverting ileostomy was performed. After covering the exteriorized colonic segment and polyglycolic sutures with gauze to ensure their stability, the first step was completed. The patient was transferred to the intensive care unit, and on the first postoperative day, he was discharged to inpatient service.

**Figure 2:**
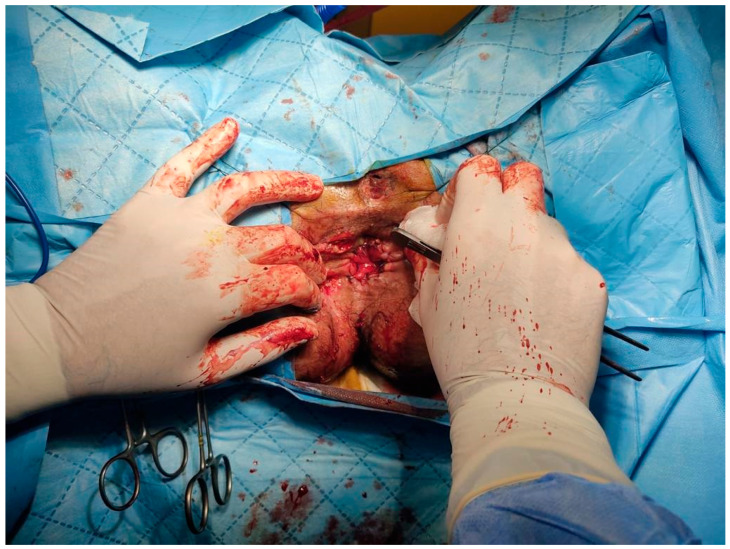
Maturated coloanal anastomosis.

Five days after the first step, the second step was completed. In the second step, the anal canal was retracted with four 0 silk sutures. Then, the exteriorized colonic segment was amputated from above the transitional zone, and coloanal anastomosis was completed by passing the pre-installed eight polyglycolic acid sutures from the colon (Figure 2).

In the postoperative period, he had rectal discharge and minimal hemorrhage. The discharge was treated with 500 mg of ciprofloxacin, and the hemorrhage was followed up without any additional treatment or intervention. In the second month after surgery, rectoscopy revealed a 2 cm long pouch on the anterior side of the neorectum from 3rd centimeter of the anal verge. The colon was edematous and hyperemic. Then, the patient was treated with 500 mg of ciprofloxacin and 500 mg of metronidazole twice a day for 20 days, in addition to four doses of infliximab. Following the treatment, the symptoms of the patient regressed. A rectoscopy was done in the seventh postoperative month, and the pouch on the anterior side of the anastomosis was closed. No ulcerative lesion or fistula was seen. Purulent discharge from the anal canal was noted. In the 10th postoperative month, an abscess was seen in contrast-enhanced lower abdomen computed tomography, and medical therapy was initiated. The patient was treated with 1000 mg of amoxicillin-clavulanic acid and 500 mg of metronidazole three times a day for 10 days. In the following 15 days, 500 mg of ciprofloxacin twice a day was added, and amoxicillin-clavulanic acid was stopped.

**Figure 3:**
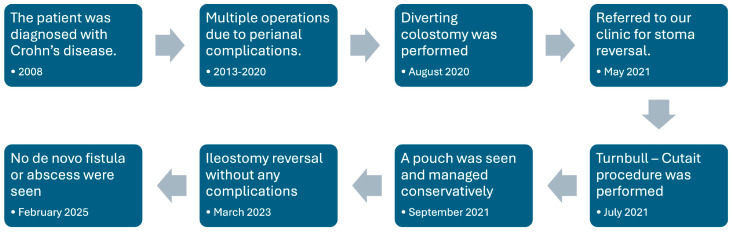
Timeline of the management of the patient.

In the 20th postoperative month, rectoscopy showed no active inflammation, abscess, or fistula. The anastomosis and neorectum were found suitable for the ileostomy reversal by the gastroenterologist specialized in pelvic floor diseases and physiotherapy, and the patient was referred to our general surgery clinic for the ileostomy reversal. The ileostomy was closed by hand-sewn end-to-end anastomosis with silk sutures in March 2023. He was discharged without any complication on the fourth postoperative day. Pelvic floor physiotherapy was suggested to the patient. In the second postoperative month, a pilonidal abscess was reported, and the patient was treated with 1000 mg of amoxicillin-clavulanic acid twice a day for 7 days. In the 42nd postoperative month, no de novo anal fistula and complications were reported (Figure 3).

## 3. Discussion

This is a case report of a patient with perianal CD treated with the TC pull-through coloanal anastomosis. The management of perianal CD and its septic complications, including perianal abscess and fistula, is a widely researched area [1,4,5]. With the development of biological agents and immunomodulators, the radical surgery requirement is decreasing day by day [4]. Although combining medical and mechanical treatment options is promising in the management of perianal CD, more than 90% of the patients undergo at least one surgical procedure in a wide range of majority [12]. The vast majority of these interventions are related to perianal abscess drainage accompanied by fistulas.

In CD patients, delayed initiation of anti-TNF alpha therapy is one of the major factors shortening surgery-free survival. In a retrospective observational study conducted among Japanese patients, it was found that Crohn’s patients who started anti-TNF alpha therapy had a significantly lower risk of undergoing surgery (OR = 0.0446, *p* < 0.001) [13]. In our case, the patient received only mesalazine treatment for the first 5 years and began biological therapy only after developing perianal complications at the end of the 5-year period. However, the delayed initiation of biological agents and the insufficient suppression of the inflammatory response during this time adversely affected the progression of the disease [14].

The management of CD is a patient-centric complex process. Different approaches are available to increase the QoL and decrease the disease-related comorbidities [15]. Various surgical approaches can be chosen in every step of complex fistula management. In addition to non-cutting seton placement, fistulotomy, endorectal flap, and mesenchymal stem cell therapy are other options in selected patient groups [15]. As in our patient, fistulizing CD patients require multiple seton placements and abscess drainages due to poor wound healing and the biology of the disease. Every intervention in these patients brings its own complication risk together. Recurrent trauma to the perianal region which is already covered by an inflamed mucosa layer can cause de novo fistulas or anal stricture due to fibrotic healing. In these cases, temporary fecal diversion should be taken into consideration to improve QoL and decrease possible complications. Until 2020, our patient was followed up with interventions regarding fistulas and abscesses, but his recurrent symptoms and incontinence decreased his QoL and complicated the management of symptoms.

Temporary fecal diversion is a widely applied choice to decrease the bacterial load from the inflamed perineum [6]. Following the diversion, 88.9% of these patients with perianal involvement require total proctectomy and permanent stoma, which is 7.5 times higher than patients without perianal involvement [16,17]. Pull-through anastomosis offers anal canal preserving surgery without a permanent stoma to CD patients with isolated rectal involvement.

Anal stricture is another challenging complication of perianal CD due to chronic and long-term inflammation of the tissue and loss of function of the anal muscles. It is associated with the severity of the disease and can be used as a strong prognostic factor for bowel continuity in the future [5]. Anal stricture-related comorbidities are important reasons for fecal diverting in patients with CD, and outcomes are poor even patients’ infection-related morbidities are regressed [18]. Gu et al. [17] reported that disease limited to perianal disease is a favorable factor that increases the rate of stoma reversal up to 50%. As in our patient, patients with disease limited to the perianal region and distal rectum can be appropriate candidates for temporary fecal diversion.

The permanent stoma is associated with a decrease in the QoL for patients. Even young patients, such as in our case, with permanent stomas, have more potential to adapt to the condition compared to older patients, but their QoL never reaches the same level as a healthy person [19]. Patients with a stoma are prone to isolate themselves from social gatherings, experience difficulty tolerating stressful and challenging conditions that they used to tolerate, and experience self-image anxiety, which results in reduced sexual activity [20,21]. In our case, the patient’s stoma was reversed in the 20th postoperative month following the CD-related medical therapy and an adequate pelvic floor physiotherapy program. In the management of these patients, the end stoma should always be the end-resort option, and sphincter preserving options should be evaluated to maximize patients’ QoL and avoid permanent diversion.

TC is a historical surgical approach first described for Hirschsprung and Chagas diseases [8,9]. Today, this approach is being used for very low rectal cancer surgery as an alternative to coloanal anastomosis. In selected patients, it is superior to immediate coloanal anastomosis in both short-term and long-term aspects [22]. Remzi et al. [23] reported that TC is a promising approach for treating patients with complex fistulas, irradiated pelvis, or pelvic inflammation history. TC patients had less anastomotic leakage and abscess formation (*p* = 0.0048 and *p* = 0.0043, respectively). Thus, TC can take priority over immediate coloanal anastomosis in patients with a pelvic infection history, such as in our case. It allows preserving the anal canal with less risk of anastomotic leakage and abscess formation.

The Turnbull–Cutait procedure was used as an option to end stoma in complex pelvic fistulas in patients with CD. A study by Lavryk et al. [24] has reported that 26 patients underwent TC for rectovaginal fistula (RVF). A total of 7.7% of the patients had anastomotic disruption and ended with permanent stoma. In our case, our patient’s anastomotic disruption was minimal and managed conservatively without the need for a permanent stoma. Ensuring a well-perfused colonic stamp is crucial to prevent tissue necrosis and anastomotic disruption. Four out of the cases were CD-related RVF. The success rate of the TC procedure was reported 75% in this highly selected group of patients.

## 4. Conclusions

In conclusion, TC can be an option to end stoma in highly selected patients with isolated perianal CD. However, it is crucial to recognize that only a small portion of the CD patients who have exhausted all other medical and surgical alternatives may be eligible to undergo this procedure. TC has the potential of being a safe, comfortable, and promising solution for carefully selected patients following prospective trials with larger series. It is essential to offer this surgery following extensive follow-up and comprehensive evaluations, considering that the isolated perianal involvement affects only a small portion of CD patients and an end stoma may still be required after the TC procedure.

## Data Availability

Data are contained within the article.

## Abbreviations

CD	Crohn’s disease
TC	Turnbull–Cutait
QoL	quality of life
